# Effect of Autophagy Inhibitors on Radiosensitivity in DNA Repair-Proficient and -Deficient Glioma Cells

**DOI:** 10.3390/medicina58070889

**Published:** 2022-07-02

**Authors:** Tareq Saleh, Homood M. As Sobeai, Ali Alhoshani, Khalid Alhazzani, Mashal M. Almutairi, Moureq Alotaibi

**Affiliations:** 1 Department of Basic Medical Sciences, Faculty of Medicine, The Hashemite University, Zarqa 13115, Jordan; tareq@hu.edu.jo; 2 Department of Pharmacology and Toxicology, College of Pharmacy, King Saud University, Riyadh 11451, Saudi Arabia; hassobeai@ksu.edu.sa (H.M.A.S.); ahoshani@ksu.edu.sa (A.A.); kalhazzani@ksu.edu.sa (K.A.); mmalmutairi@ksu.edu.sa (M.M.A.)

**Keywords:** DNA damage, autophagy, senescence, glioblastoma, DNA-PKcs, radiation

## Abstract

*Background and Objectives*: The development of radioresistance is a fundamental barrier to successful glioblastoma therapy. Autophagy is thought to play a role in facilitating the DNA repair of DNA damage foci in radiation-exposed tumor cells, thus, potentially contributing to their restoration of proliferative capacity and development of resistance in vitro. However, the effect of autophagy inhibitors on DNA damage repair is not fully clear and requires further investigation. *Materials and Methods*: In this work, we utilized M059K (DNA-PKcs proficient) and M059J (DNA-PKcs deficient) glioma cell lines to investigate the role of autophagy inhibitors in the DNA repair of radiation-induced DNA damage. Cell viability following radiation was determined by trypan blue exclusion in both cell lines. Cell death and autophagy assays were performed to evaluate radiation-induced cell stress responses. DNA damage was measured as based on the intensity of phosphorylated γ-H2AX, a DNA double-stranded breaks (DSBs) marker, in the presence or absence of autophagy inhibitors. *Results*: The cell viability assay showed that M059J cells were more sensitive to the same dose of radiation (4 Gy) than M059K cells. This observation was accompanied by an elevation in γ-H2AX formation in M059J but not in M059K cells. In addition, the DAPI/TUNEL and Senescence-associated β-galactosidase (SA-β-gal) staining assays did not reveal significant differences in apoptosis and/or senescence induction in response to radiation, respectively, in either cell line. However, acridine orange staining demonstrated clear promotion of acidic vesicular organelles (AVOs) in both cell lines in response to 4 Gy radiation. Moreover, DNA damage marker levels were found to be elevated 72 h post-radiation when autophagy was inhibited by the lysosomotropic agent bafilomycin A1 (BafA1) or the PI3K inhibitor 3-methyl adenine (3-MA) in M059K cells. *Conclusions*: The extent of the DNA damage response remained high in the DNA-PKcs deficient cells following exposure to radiation, indicating their inability to repair the newly formed DNA-DSBs. On the other hand, radioresistant M059K cells showed more DNA damage response only when autophagy inhibitors were used with radiation, suggesting that the combination of autophagy inhibitors with radiation may interfere with DNA repair efficiency.

## 1. Introduction

Radiotherapy is frequently utilized in combination with other anticancer modalities to treat a variety of cancers. Nevertheless, the response of cancer cells to radiation is variable and the development of resistance to radiation is often encountered [1]. For example, the use of radiation, either post-surgically or along with chemotherapeutic agents, improves the potency of breast, head, and neck cancer therapies [2,3]. However, in other types of cancers, such as glioblastoma and lung cancer, the rate of radioresistance is higher, and thus, cancer recurrence, that usually occurs within few years after completion of therapy, is more common [4,5,6]. Unfortunately, recurrent cancer is more aggressive and its response to conventional therapy is poor [7,8]. The mechanism of radiation-induced cytotoxicity is largely dependent on inflicting DNA damage which may then lead to cell death (apoptosis) [9,10]. However, it has been shown that DNA damage can also induce several other modes of cell stress responses, such as senescence, autophagy, and mitotic catastrophe [10,11,12,13]. 

The exact role of autophagy in mediating the effect of chemotherapy and radiation in cancer cells is still controversial [14,15]. In some tumor models, autophagy plays a pro-survival role, where autophagy induction in response to therapy exposure protects cells against cell death, which reflects the classical cytoprotective function of autophagy [16]. In contrast, under certain conditions, treatment of tumor cells with chemotherapy or radiation triggers a cytotoxic form of autophagy, where autophagy inhibition reduces the extent of cell death in a tumor cell population [17]. This dual contribution of autophagy to cell survival has been demonstrated in different models of glioblastoma exposed to various cancer chemotherapies [18]. The mechanisms that precisely dictate the functional contribution of therapy-induced autophagy remain unclear. 

It has been established that autophagy is promoted as a consequence of the exposure to DNA damaging agents, including ionizing radiation [19]; however, the crosstalk between autophagy and the DNA repair system is unclear. For example, inhibition of autophagy by the lysosomotropic agent bafilomycin A1 (BafA1) can sensitize glioma cells to temozolomide by increasing the rates of apoptosis induction [20], and interference with autophagy is associated with an impaired DNA repair system [21]. In addition, 6-thioguanine-induced autophagy exhibited a pro-survival function in human colorectal and endometrial tumor cells, which points towards a protective role of autophagy against DNA damage [22]. Moreover, previous evidence indicated a role for autophagy in regulating DNA repair through the activation of cell cycle checkpoints and an influence on homologous recombination repair (HRR) [23]. Furthermore, the link between DNA damage and autophagy was shown to be mediated via PARP-1, in which ATP and (NAD+) are depleted, leading to the activation of cytoprotective autophagy [24].

A recent publication has summarized the findings of several pre-clinical and clinical reports on the implementation of autophagy modulators for the treatment of glioblastoma and concluded that it is still unclear whether autophagy inhibition or induction are effective anticancer approaches [25]. As such, further investigation of the actual contribution of autophagy to the response to cancer treatments is required. The main aim of this study is to understand the role of autophagy inhibitors in mediating the DNA-damaging effects of radiation in glioblastoma cells and address whether radiation-induced autophagy plays a cytoprotective function, in which autophagy inhibition can sensitize glioblastoma cells to radiation. In order to address this question, M059K (DNA-PKcs proficient or expressing) and M059J (lack the expression of DNA-PKcs) glioma cell lines were used. DNA-PKcs is a catalytic subunit that is involved in non-homologous end joining (NHEJ) and forms a holoenzyme called DNA-PK when bound to KU subunits during the activation of NHEJ to initiate DNA repair [26]. Thus, loss of DNA-PKcs function is associated with defective repair of DNA double-stranded breaks (DSBs). In addition, the ataxia telangiectasia mutated (ATM) is barely detectable in extracts from M059J cells but has normal expression levels in M059K cells [27,28,29]. Both cell types were exposed to radiation and evaluated for senescence, apoptosis, and autophagy. While autophagy was found to be promoted in both cell lines, there was no evidence of apoptosis and senescence induction. The promotion of autophagy was accompanied by G_2_ arrest in the M059J cells but not in the M059K cells. Pharmacological inhibitors of autophagy, such as BafA1 and 3-methyladenine (3-MA), were used to further assess DNA repair kinetics following radiation. The extent of phosphorylated H2AX (γ-H2AX) formation, a marker of DNA DSBs, was significantly higher when autophagy was pharmacologically blocked in M059K (DNA-PKcs-expressing) cells. Consistently, cell viability assays showed that M059K (DNA-PKcs-deficient) cells were significantly sensitized to radiation when autophagy inhibitors were applied. Taken together, our data suggest that pharmacological interference with autophagy may delay DNA repair and radiosensitize glioma cells.

## 2. Materials and Methods

### 2.1. Cell Culture

M059K (DNA-PKcs-expressing human glioma cells) and M059J (DNA-PKcs deficient human glioma cells) were kindly provided from the Dr. Lawrence Povirk laboratory at Virginia Commonwealth University. Both cell lines were cultured in RPMI 1640 medium supplemented with 5% fetal bovine serum, 5% bovine calf serum, 2 mM L-glutamine, and penicillin/streptomycin (GIBCO Life Technologies, Gaithersburg, MD, USA). Once thawed off, cells were incubated in a 75 cm^2^ flask at 37 °C, under a humidified environment of 5% CO_2_, and underwent few passages before the initiation of experiments. Cells were exposed to the indicated treatment, under the same conditions, in every assay. Irradiation treatments were undertaken at a dose of 4 Gy using the Gammacell Exactor 40 (Theratronics Ltd., Ottawa, ON, Canada). The dose of 4 Gy radiation was selected based on its ability to induce an autophagic response in glioblastoma cell lines in previous studies [30,31].

### 2.2. Cell Viability

Cells were plated in 6-well plates (generally 200,000 cells/well) and allowed to adhere overnight. The next day, the cells were irradiated, and the number of viable cells was counted at indicated time points for 5 days. In the case when an autophagy inhibitor was utilized (BafA1 or 3-MA) with radiation treatment, cells were pre-exposed to the drug 3 h prior to radiation and then the drug-containing medium was removed 24 h after radiation [32]. At each time point, media were removed, and cells were washed one time with 1X phosphate buffered saline (PBS). Trypsin (0.25%) was added for 5 min to detach the cells and then deactivated by adding serum-containing fresh media to make up a total of 1 mL of cell suspension. Cells were collected in 1 mL conical tubes (Eppendorf Inc., Westbury, NY, USA) and 10 µL of cell suspension was added to trypan blue (0.4%), placed onto a hemocytometer (Hausser Scientific, Horsham, PA, USA), and counted manually under a light microscope.

### 2.3. Assessment of Autophagy by Acridine Orange Staining

Acridine orange (Sigma-Aldrich, A6014, St. Louis, MO, USA) staining solution was prepared in PBS at a ratio of (1:10,000) to reach to a final concentration of 100 ng/mL (prepared in the dark), added to cells, and incubated for 15 min at 37 °C. Plates were washed again with 1X PBS and fresh medium was added. Images were taken using an Olympus 1X 70 microscope and an Olympus SC 35 camera, as performed in previous publications [32,33].

### 2.4. Evaluation of Senescence by β-Galactosidase Staining

For the microscopic visualization of senescent cells, senescence-associated β-Galactosidase (SA-β-gal) staining was performed [34]. Cells were washed once with 1X PBS and fixed with a mixture of 2% formaldehyde and 0.2% glutaraldehyde for 5 min, then incubated overnight in a CO_2_-free incubator at 37 °C and monitored pH 6.0 in a staining solution (1 mg/mL 5-bromo-4-chloro-3-inolyl-β-galactosidase in dimethylformamide (20 mg/mL), 5 mM potassium ferricyanide, 150 mM NaCl, 40 mM citric acid/sodium phosphate, 2 mM MgCl_2_). Cells were then washed with 1X PBS and pictures were obtained via a bright field Olympus 1X 70 microscope and an Olympus SC 35 camera. For quantification of senescent cells, β-galactosidase detection by flow cytometry post-treatment was undertaken [35]. Live cells were incubated for 1 h in 100 nM of bafilomycin A1 in order to allow for pH adjustment. A C_12_FDG-containing medium (33 µM) was added to each well for 2 h, media were washed out twice with PBS for ~30 s per wash. Cells were then collected and centrifuged at 1500 rpm for 5 min at 4 °C. Cells were resuspended in ice-cold PBS and analyzed by flow cytometry. C_12_FDG is a substrate that undergoes hydrolysis by upregulated β-galactosidase enzymes and becomes fluorescent at wavelengths of 500–510 nm [35].

### 2.5. Determination of γH2AX Intensity as a Marker of DNA Damage

The measurement of DNA damage was performed as previously described [32]. Briefly, both cell lines were seeded in 4-chamber cover glass slides and left overnight to allow for attachment. The next day, cells were exposed to radiation and then fixed with 90% ethanol and maintained at −20 °C until the day of the experiment. Cells were then centrifuged at 3000 rpm for 5 min and resuspended in 1% BSA for 30 min. The Gamma-H2AX antibody (BD Pharmingen) was added to the cells in a dilution of (1:200) and incubated at room temperature for 1 h. Cells were then analyzed by flow cytometry at an excitation wavelength of 488. Raw data were normalized according to the intensity of control samples (normalized mean intensity ¼ intensity of the sample/the intensity of the corresponding control sample within the same experiment).

### 2.6. Evaluation of Apoptosis

Cells were treated in 6-well plates and collected on cytospin slides. For fixation, cells-containing slides were kept in 4% formaldehyde in PBS for 10 min, washed in PBS, and fixed again with acetic acid (1:2 in ethanol). For the TUNEL (terminal deoxynucleotidyl transferase dUTP nick end labeling) assay, non-specific binding sites were blocked with BSA (1 mg/mL for 30 min), washed twice with PBS for 5 min each, and incubated at 37 °C under a humidified environment of 5% CO_2_ with an enzyme mixture (terminal transferase, 25 mM CoCl_2_, fluorescein-12dUTP) for 1 h to facilitate the enzymatic reaction [36]. Slides were washed with PBS for 5 min and DAPI (1:1000 dilution) was added. Images were obtained using a fluorescent microscope.

### 2.7. Cell Cycle Analysis

At the day of experiment, cells were harvested and centrifuged twice at 1500 rpm. Every time, supernatant was removed, and pellets were resuspended again in 1X PBS. Cells were gently resuspended in 0.2 mL of PBS and fixed with 1.8 mL of cold 70% ethanol solution. Cells were then washed with PBS prior to the addition of a staining solution (0.1% (*v*/*v*) Triton-X-100 in 10 mL PBS, 2 mg of DNase free RNase A, and 0.2 mL of the propidium iodide stock (1 mg/mL) 2 h prior to being examined by flow cytometry [37].

### 2.8. Statistical Analysis

Statistics were performed using student test (*t*-test). The significance of group values was determined based on a *p* value of less than 0.0.5 (*p* < 0.05). 

## 3. Results

### 3.1. Differential Response of M059K and M059J Cell Lines to Radiation

**Figure 1:**
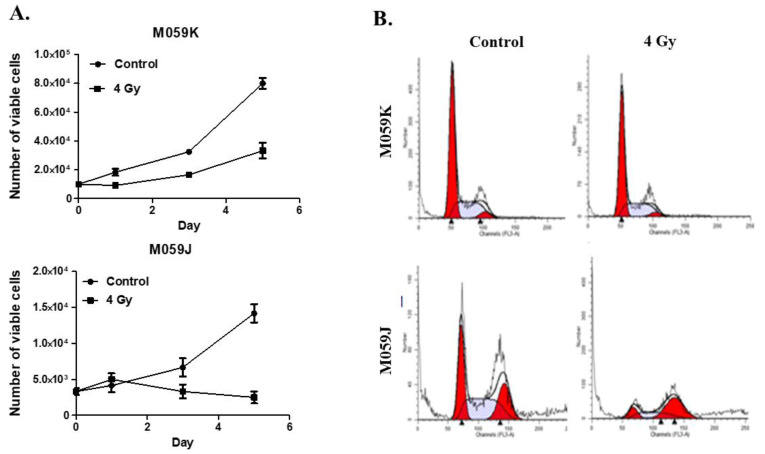
Radiation induces growth inhibition in repair-deficient cells but not in repair-proficient cells. (**A**) Exposure to radiation induces growth inhibition followed by delayed cell death in repair-deficient M059J cells (lower panel) but only delayed growth in repair-proficient M059K (upper panel). On day 0, cells were irradiated with 4 Gy and counted on days 0, 1, 3, and 5. Y axis represents the number of viable cell number as measured by trypan blue exclusion. (**B**) Cell cycle analysis showed that M059J cells were still growth-arrested at the G_2_/M phase of the cell cycle after 72 h of radiation, whereas M059K recovered their growth potential, showing a cell cycle distribution comparable to control, vehicle-treated cells. Consistently, cell viability results showed clear recovery in M059K cells but not in M059J cells, suggesting that deficiency in DNA repair due to lack of DNA-PKc function leads to prolonged growth inhibition upon radiation. Error bars represent standard error of the mean (SEM) in A.

Previous studies have shown that M059J cells are more sensitive to low doses of radiation than M059K cells [31]. In our initial studies, we used M059K (DNA-PKcs-expressing) and M059J (lack the expression of DNA-PKcs) human glioma cells. To first characterize the sensitivity of these cells to radiation, we monitored the cell viability of each cell line over a period of 5 days post-irradiation (4 Gy). After radiation exposure at 4 Gy, M059J cells underwent a transient growth arrest followed by delayed cell death, while M059K cells showed proliferative recovery 72 h post-treatment (Figure 1A). This observation is consistent with previous studies which indicated that reduced DNA repair capacity increases sensitivity to radiation [31]. Since M059K showed complete recovery 72 h post-treatment, whereas M059J continued to be in a state of growth arrest based on the number of viable cells shown in the growth curves (Figure 1A), we expected that M059J succumbed to a more stable phase of cell cycle arrest due to defective DNA repair. To confirm that hypothesis, we ran a cell cycle analysis on both cell lines after radiation and found that M059J cells were arrested at the G_2_ phase 72 h post-treatment, whereas M059K showed a cell cycle distribution similar to control, not-radiated cells (Figure 1B).

### 3.2. Induction of Autophagy, but Neither Apoptosis or Senescence, by Ionizing Radiation in DNA-PKcs-Expressing and DNA-PKcs-Not Expressing Cells

**Figure 2:**
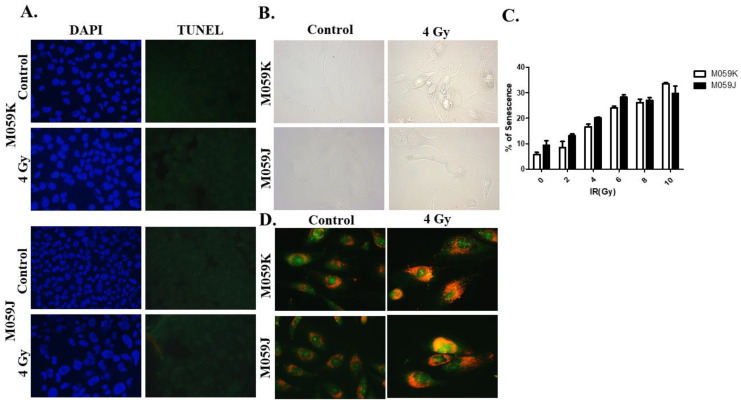
Promotion of autophagy and absence of senescence and apoptosis upon exposure to radiation in M059K and M059J cells. (**A**). Cells were treated with a dose of 4 Gy and stained with DAPI (left) and TUNEL (right) to determine the portion of cells that undergo apoptosis upon radiation. Irradiated cells (bottom) images show no TUNEL-positive cells in both cell lines suggestive of minimal apoptosis induction. (**B**). SA-β-galactosidase upregulation was monitored in M059K and M059J cells to evaluate senescence markers post-radiation. Images show minimal expression of SA-β-galactosidase in both cell lines. (**C**). Quantification of senescence in M059K and M059J cells by flow cytometry-based measurement of the SA-β-galactosidase fluorogenic surrogate C_12_FDG. (**D**). Both cell lines were exposed to 4 Gy and stained with acridine orange 72 h post-treatment. Treated cells showed increased accumulation of acidic vacuoles and enlargement of cell morphology. All images were captured under 200x magnification.

We then wanted to investigate which form of cell stress response is induced following irradiation in both cell lines. We were anticipating that M059J cells, but not M059K cells, would show markers of apoptosis due to their relative sensitivity to radiation, as previously shown in Figure 1. Intriguingly, our DAPI/TUNEL staining results revealed that apoptosis was only minimally induced in either cell line (Figure 2A), suggesting that M059J cells might have undergone an alternative form of cell death/cell cycle arrest. We then investigated whether radiation-induced growth arrest was associated with the induction of senescence [38], since residual surviving cells were observed in irradiated M059J cells. Interestingly, our β-galactosidase staining images did not demonstrate upregulation of SA-β-galactosidase in either cell line (Figure 2B). Quantification of senescent cells by flow cytometry indicated that senescence was induced minimally (less than 25% under 4 Gy radiation) and no differences were found between DNA-PKcs-expressing and DNA-PKcs-deficient glioblastoma cells (Figure 2C). We next sought to evaluate whether autophagy was induced in both cell lines in response to irradiation. Although the basal level of acidic vesicular organelles (AVOs) was high in control, non-radiated cells, irradiated cells showed an enlargement in cell size and increased accumulation of acidic vacuoles, which is suggestive for the induction of autophagy in both cell lines (Figure 2D). 

### 3.3. Effects of Autophagy Inhibitors on Radiosensitization and DNA Repair Capacity

**Figure 3:**
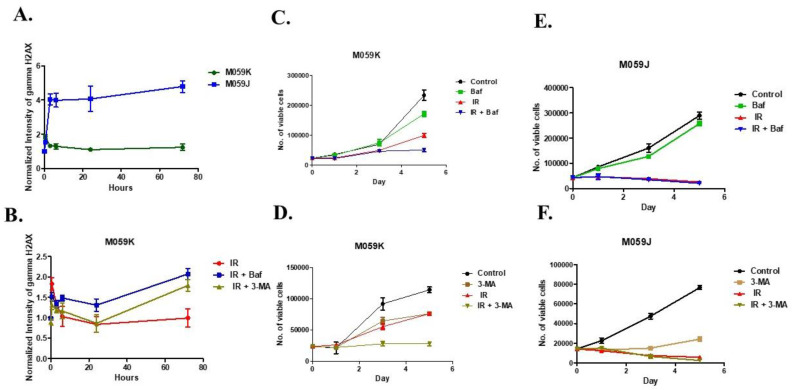
Autophagy inhibitors affect responses to radiation and interrupt DNA repair. (**A**). M059K and M059J cells were exposed to 4 Gy and the extent of γ-H2AX formation was evaluated by performing flow cytometry at 0, 30 min, 3, 6, 24, and 72 h. (**B**). M059K cells were pretreated with Bafilomycin A1 (50 nM) or 3-MA (5 mM) to inhibit autophagy and monitored γ-H2AX formation in irradiated cells at 0, 30 min, 3, 6, 24, and 72 h. (**C**). M059K cells were pretreated with Bafilomycin A1 (50 nM) and irradiated with 4 Gy on day 0 and counted on days 1, 3, and 5. (**D**). M059K cells were pretreated with 3-MA (5 mM) and irradiated with 4 Gy on day 0, and counted on days 1, 3, and 5. (**E**). M059J cells were pretreated with Bafilomycin A1 (50 nM) and irradiated with 4 Gy on day 0, and counted on days 1, 3, and 5. (**F**). M059J cells were pretreated with 3-MA (5 mM) and irradiated with 4 Gy on day 0, and counted on days 1, 3, and 5. Error bars represent standard error. For all these figures “control” indicates vehicle (DMSO)-treated cells that were not exposed to radiation, Baf, or 3-MA.

We next sought to investigate the effect of autophagy inhibitors on radiated M059K and M059J glioma cell lines, namely the lysosomotropic agent BafA1 and the PI3K inhibitor 3-MA. The capability of cells to recover suggested that DNA repair is likely to be functional in the autophagic M059K cells but not the M059J cells. The goal of the next experiments was to determine whether the blockade of autophagy using these compounds would promote DNA damage-induced cell death and the accumulation of DNA damage foci, primarily in the resistant M059K human glioma cells. To address this question, M059K and M059J cells were treated with radiation (4 Gy) and allowed to undergo repair for 3 days. Repair foci were measured after 30 min, 3, 6, 24, and 72 h based on γ-H2AX immunostaining. Three days later, the level of γ-H2AX remained high in M059J cells but not in M059K cells (Figure 3A). In addition, the intensity of γ-H2AX was further assessed in M059K cells when pre-treated with BafA1 (50 nM) and 3-MA (5 mM) for the same exposure period. Interestingly, interference with autophagy using BafA1 and 3-MA in M059K cells led to an increase in γ-H2AX levels when cells were exposed to 4 Gy of ionizing radiation in 72 h. However, the intensity of γ-H2AX was insignificant between groups by 24 h, suggesting that, despite their proficiency in DNA repair, these cells were still generally showing a persistence of initial DNA damage during the inhibition of autophagy after 72 h (Figure 3B). Accordingly, it was important to monitor cell viability over time while co-treating with autophagy inhibitors in both cell lines. Consistent with our observation in Figure 3B, both inhibitors have significantly radiosensitized M059K cells, whereas no such sensitivity was found with M059J cells (Figure 3C–F). Collectively, our data suggest that autophagy inhibitors may have an effect on the DNA damage repair system in cells exposed to genotoxic stress, but the role of autophagy may differ according to the status of DNA repair.

## 4. Discussion

Several studies have indicated that autophagy is involved in the chemoresistance of tumor cells to DNA-damaging agents [39,40]. The link between autophagy and DNA repair remains unclear [41,42], but the role of autophagy is to provide an avenue whereby cells manage to repair DNA damage during a state of growth arrest or to be directly involved in facilitating the DNA repair machinery. Therefore, in this work, we proposed that autophagy inhibitors would lead to a delay in the repair of radiation-induced DNA double-strand breaks (DSBs), potentiating the DNA damaging efficacy of radiation. We assessed the ability of cells to repair the damaged foci through the measurement of the extent of phosphorylated H2AX (γ-H2AX) formation after pretreatment of cells with pharmacological inhibitors of autophagy. The outcomes of autophagy inhibition, using both early (3-MA) and late (BafA1) autophagy inhibitors, suggested a delay in the repair of DNA DSBs. First, we found that M059K cells showed faster reduction in γ-H2AX formation compared to M059J cells in a time-dependent manner when treated with radiation only. Second, co-treatment with autophagy inhibitors led to a delay in the repair of DSBs over time. Indeed, more studies are needed utilizing genetic silencing of autophagy-regulating genes such as ATG5, ATG7, and/or Beclin-1 to confirm the role of autophagy in mediating radiation-induced DNA damage.

Previous evidence has supported the premise that autophagy inhibition leads to impairment of successful DNA damage repair. For example, in colorectal cancer cells, the suppression of key autophagy regulators, such as members of the ATG family or Beclin-1, have been shown to interfere with the repair of radiation-induced DNA damage [43]. Utilization of the classical lysosomotropic agent chloroquine (CQ) in combination with radiation inhibits autophagy and delays DNA damage repair, which significantly interferes with the clonal survival of glioma cells and drives them into apoptotic cell death [44]. In recent evidence, the inhibition of cytoprotective autophagy induced by the exposure of glioma cells to radiation and DNA damage significantly enhanced the antitumor potential of radiation [45]. Collectively, data from the literature support the premise that autophagy inhibition is likely to favor a cell death response to radiation due to failure of optimal DNA damage repair.

The connection between autophagy and DNA damage repair can be more complicated. Of relevance, human malignant glioma cells were shown to undergo autophagic cell death upon the inhibition of DNA–PKcs, a protein involved in non-homologous end-joining [31]. Furthermore, the inhibition of DNA-PKcs in resistant prostate cancer cells radiosensitized these cells by inducing autophagy [46]. It has been shown that cells lacking DNA-PKcs do not undergo apoptosis, suggesting that autophagy may play an alternative role when cells lack efficient DNA repair [47,48]. Our data showed that exposing M059K and M059J cells to radiation only (4 Gy) did not induce apoptosis which is likely to be due to the promotion of IR-induced autophagy. Our data were consistent with previous studies, suggesting that M059J cells undergo autophagic cell death in response to irradiation [17,31,49,50]. Moreover, and in agreement with our findings, utilizing low-dose radiation in both M059K and M059J cells was not associated with a significant increase in apoptosis induction, based on TUNEL assays [31]. In fact, the induction of minimal or delayed apoptosis in response to low-dose radiation was reported in U87 and T98G glioma cells [30,51], A549 non-small cell lung cancer cells [19,52], MDA-MB231 breast cancer cells [53], and HCT116 colorectal cancer cells [32]. Moreover, in a very recent report by Li et al. highlighted the differential responses to radiation in cancer cell lines, and that other cell stress mechanisms, such as autophagy and senescence, might play more important roles than apoptosis in mediating the effect of low-dose radiation in cells [54]. In the clinic, a subset of tumors, which can be large in the case of glioblastoma, develop resistance to radiation primarily due to lack of cell death induction. In fact, the response of glioblastoma to radiation is variable whereas some tumors have a poor response to radiation (based on their molecular subtyping) [55], which can be attributed to the variability (and great heterogeneity) in the expression of apoptosis regulatory pathways [56,57]. Lastly, an explanation of the difference that might be observed in the clinic is based on the clinical use of fractionated radiation, in comparison to a single dose. This recently led to the proposal to use hyofractionated radiotherapy regimens, with sufficient spacing to avoid the differential outcomes in cell stress responses to therapy [58], and/or the use of pro-apoptotic compounds to enhance the efficacy of radiotherapy [59,60].

Furthermore, we observed greater radiosensitization in autophagy-inhibited M059K cells than cells treated with radiation alone. As we found that cells exposed to autophagy inhibitors showed delayed repair, persistent DNA damage may commit cells into irreversible cell death. Lastly, while our studies are in agreement with the previous literature that demonstrates autophagy induction in glioblastoma cell lines exposed to radiation, our studies are largely based on the measurement of autophagic vacuole formation (or AVOs), and must be complemented in the future with the examination of LC3B lipidation and p62/SQSTM1 degradation [61]. Moreover, genetic knockdown of autophagy-regulating genes is an essential future effort that needs to be performed to elucidate the role of upstream autophagy inhibition in DNA repair and to rule out any possible off-target effect of the utilized pharmacological inhibitors of autophagy. It is noteworthy that despite significant difference in the protein expression levels of ATM between the M059K and M059J cell lines, that is unlikely to contribute directly to their variable radiosensitivity [28,29]; the main driver of dysfunctional DNA repair is lack of DNA-PKc in the M059J [27]. However, recent evidence has strongly linked ATM to the induction and function of autophagy in response to genotoxic stress (reviewed in depth in [62,63]). For example, ATM regulates radiation-induced autophagy through the mTOR pathway [64,65], MAPK14 pathway [66], JNK pathway [67], and PI3KIII pathway [68], and complete lack of ATM function results in the failure of autophagy induction in tumor cells [68,69,70] or switches the cell stress response to senescence [71]. Conversely, Goehe et al. showed that ATM knockdown attenuates both autophagy and senescence in response to chemotherapy [69], suggesting a more complicated role of ATM in regulating autophagy. Subsequently, the low expression levels of ATM in the M059J cells could explain, in part, the different response to autophagy inhibitors observed in comparison to the M059K cells.

In addition, we did not see an increase in SA-β-galactosidase activity in M059K or M059J glioma cells suggestive of the lack of senescence induction. It has been shown that M059K glioma cells have a mutated p53 [72], so senescence, apoptosis, and DNA repair in this system might not be well-intertwined. Although our data did not show upregulation of β-galactosidase, DNA repair-deficient cells underwent growth arrest, as evidenced by the development of growth abrogation demonstrated by the cell viability assay and the cell cycle analysis over time. The relationship between autophagy and senescence in response to DNA damaging agents is more complicated. We have shown previously that when senescence is the predominant response to radiation, the inhibition of autophagy, despite being induced in parallel to senescence, does not interfere with DNA damage repair [32]. Accordingly, when senescence is induced together with autophagy in response to radiation, it becomes more challenging to determine which process is likely to dictate the ultimate cell fate. This complexity has been proposed by Filippi-Chiela et al., who demonstrated the induction of autophagy, followed by the induction of senescence, in glioma cells exposed to temozolomide, as is the case of many other reports in the literature [73]. However, at the single-cell analysis level, Filippi-Chiela et al. showed that autophagy and senescence were induced in a significantly heterogeneous manner despite being triggered simultaneously in mass cultures [73]. Interestingly, the pharmacological suppression of autophagy triggered apoptosis but reduced the expression of senescence markers, while on the other hand, enhancement of autophagy resulted in more accelerated senescence, demonstrating that DNA damage-induced autophagy is likely to be cytoprotective. Overall, our model provided a unique opportunity to understand the relationship between autophagy and DNA damage repair since the dose of radiation utilized resulted in autophagy induction in isolation from senescence or apoptosis.

## 5. Conclusions

In conclusion, we show that both cell lines, under radiation, accumulate acidic vacuoles suggestive of autophagy induction without showing markers of apoptosis and/or senescence, and undergo proliferative recovery following a period of growth arrest. This suggests that tumor cells that might develop autophagy have the capacity to re-enter into the cell cycle and retain their proliferative capacity, probably through enhancement of DNA repair. The extent of the DNA damage response was more pronounced in the DNA-PKcs deficient cells, which demonstrated their inability to repair the newly formed DSBs. However, radioresistant M059K cells showed more DNA damage response when autophagy inhibitors were combined with radiation, indicating that autophagy inhibitors may interfere with DNA repair efficiency. Since the inhibition of autophagy may initially sensitize glioblastoma cells via an increased DNA damage response, this strategy appears to interfere with proliferative recovery, which is likely to contribute to disease recurrence and therapy resistance. Overall, our work provides evidence that using autophagy inhibitors is likely to enhance the DNA damaging potential of radiotherapy and thus could serve as means to prevent the development of radioresistance in glioblastoma.

## Data Availability

Not applicable.
